# Cationic Surface Charge Engineering of Recombinant Transthyretin Remarkably Increases the Inhibitory Potency Against Amyloid β-Protein Fibrillogenesis

**DOI:** 10.3390/molecules29215023

**Published:** 2024-10-24

**Authors:** Xiaoding Lin, Ting Xu, Wenqi Hou, Xiaoyan Dong, Yan Sun

**Affiliations:** Key Laboratory of Systems Bioengineering and Frontiers Science Center for Synthetic Biology (Ministry of Education), Department of Biochemical Engineering, School of Chemical Engineering and Technology, Tianjin University, Tianjin 300350, China; linxiaoding@tju.edu.cn (X.L.); xuting77@tju.edu.cn (T.X.); hwq@tju.edu.cn (W.H.); d_xy@tju.edu.cn (X.D.)

**Keywords:** Alzheimer’s disease, amyloid β-protein, amyloidosis, transthyretin, recombinant protein, inhibitors

## Abstract

The deposition of amyloid β-protein (Aβ) in the brain is the main pathogenesis of Alzheimer’s disease (AD). The development of potent inhibitors against Aβ aggregation is one of the effective strategies to combat AD. Endogenous transthyretin (TTR) can inhibit Aβ fibrillization via hydrophobic interactions, but its weak inhibitory potency hinders its application in AD therapy. Here, different recombinant TTRs were designed by cationic surface charge engineering. Compared with TTR, all positively charged recombinant TTRs showed enhanced capability in inhibiting Aβ aggregation, especially the recombinant protein obtained by mutating the acidic amino acid in TTR to arginine (TTR-nR) exhibited excellent inhibitory effect. Among them, TTR-7R remarkably increased the inhibitory potency against Aβ, which could effectively inhibit Aβ_40_ fibrillization at a very low concentration (0.5 μM). In addition, TTR-7R increased cultured cell viability from 62% to 89%, scavenged amyloid plaques in AD nematodes, and prolonged nematode lifespan by 5 d at 2 μM. Thermodynamic studies demonstrated that TTR-7R, enriching in positive charges, presented hydrophobic interactions and enhanced electrostatic interactions with Aβ_40_, leading to a significantly enhanced inhibitory capacity of TTR-7R. The research provided insights into the development of efficient recombinant protein inhibitors for AD treatment.

## 1. Introduction

Alzheimer’s disease (AD) is a devastating and fatal neurodegenerative disorder, accounting for approximately 70–80% of dementia cases [1,2]. The main clinical manifestations of AD include progressive loss of memory and thinking skills, behavioral disturbances, and personality changes [3,4]. The number of AD patients is increasing every year, seriously affecting human health and imposing a considerable economic burden on society [5,6]. Although the causes of AD are quite complicated, overwhelming evidence indicates that the abnormal accumulation and deposition of amyloid β-protein (Aβ) in the brain is the main hallmark of AD [7]. Aβ aggregation leads to a series of pathological changes in the brain of AD patients, such as synaptic loss, inflammation, oxidative stress, intracellular ion channel disruption, phosphorylation of tau protein, and eventual neuronal cell death [8,9]. Therefore, the design and development of potent Aβ aggregation inhibitors are considered a promising breakthrough for the treatment of AD.

In the field of drug discovery, various agents targeting Aβ have been validated to inhibit Aβ fibrillization, including small molecules [10,11], peptides [12], proteins [13,14], and metallodrugs [15,16]. In recent years, some endogenous proteins with amyloid-interfering properties have shown potential therapeutic values as a natural way to prevent amyloid formation, including heat shock protein (such as small heat-shock protein alpha-B-Crystallin) [17], clusterin [18], apolipoprotein E [19], and transthyretin (TTR) [20]. In the regulation of the Aβ aggregation, natural proteins as molecular chaperones not only have the advantages of high stability and biocompatibility but also exhibit multifunctionality through modification [21,22]. Therefore, protein inhibitors have been widely investigated as potential inhibitors of Aβ aggregation. However, the inhibitory effect of most protein inhibitors is weak, so large doses are generally required to achieve good inhibitory effects. Previous studies have demonstrated the importance of inhibiting Aβ aggregation by modulating the surface charge of lysozyme [23] and serum albumin [13], suggesting that modification of the charge property of a protein-based inhibitor is a promising way to develop a potent protein-based inhibitor. In addition, proteins can be modified not only via chemical modification but also via genetic engineering [24]. A recombinant protein with high inhibitory potency can be readily expressed in bacteria cells, making it possible for large-scale production in a cost-effective way.

**Scheme 1:**
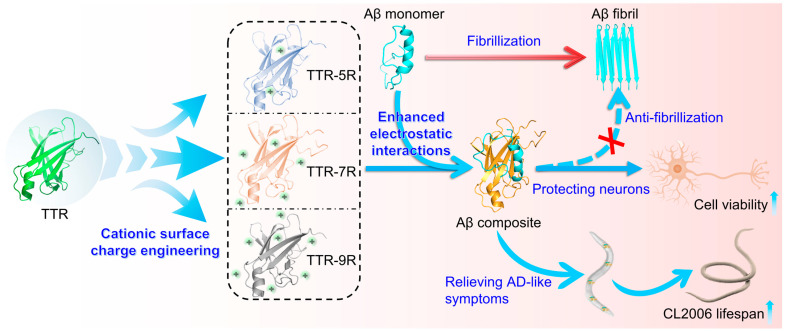
Schematic illustration of the design of recombinant TTR for the enhanced Aβ inhibition.

TTR is a 55 kDa homotetrameric protein produced mainly in the human liver and choroid plexus [25]. TTR has attracted attention because of its pathological features of aggregation and formation of amyloid fibrils [26]. TTR itself can drive amyloid formation after the dissociation of tetrameric TTR into monomers. In addition, a low-pH environment is a prerequisite for amyloid formation [27]. Different from the monomeric form of TTR dissociated from wild-type tetramers, the mutant form is a kinetically stable monomer that does not readily aggregate [28]. TTR has been reported to be the major Aβ-binding protein in cerebrospinal fluid [29], and both TTR monomer and tetramer can interact with Aβ and suppress Aβ aggregation via hydrophobic interactions [30,31]. However, the inhibitory effect of natural TTR on Aβ aggregation is quite limited [32]. Hence, this research is designed to develop recombinant TTR of high amyloid inhibitory potency by cationic surface charge engineering because TTR contains abundant charged amino acids, which affords the potential for regulating the surface charge, thereby enhancing its ability to inhibit Aβ aggregation by altering its electrostatic interactions with negatively charged Aβ. The influence of the type and number of mutated amino acids on the inhibitory capacity of the protein inhibitor was explored. All the prepared recombinant TTRs showed enhanced inhibitory capacity. The inhibitory effect of TTR-nR, obtained by mutating the acidic amino acid in TTR to arginine, was more prominent. Among them, TTR-7R showed the strongest inhibitory potency due to retained hydrophobic interactions and enhanced electrostatic interactions. Compared with TTR, positively charged TTR-7R could inhibit Aβ fibrillization and attenuate Aβ_40_-induced neuronal cytotoxicity at a very low concentration (2 μM), thereby prolonging the life of AD nematodes by 5 d (Scheme 1). This research will broaden the applications of protein inhibitors on the inhibition of Aβ aggregation and provide deep insights into the design of recombinant proteins to fight against AD.

## 2. Results and Discussion

### 2.1. Expression and Characterization of Recombinant TTR

**Figure 1:**
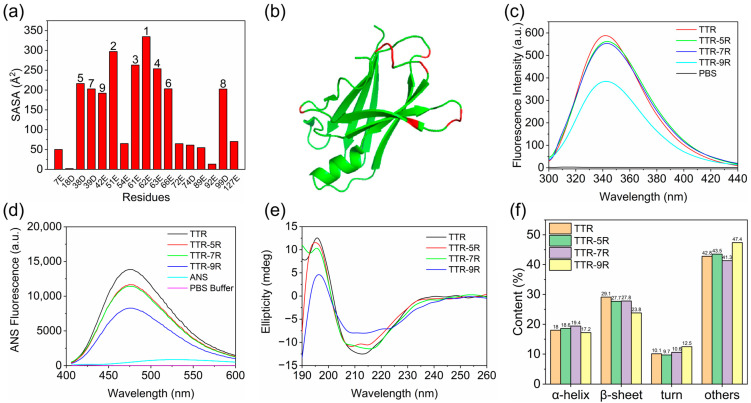
Characterization of TTR and TTR-5R/7R/9R proteins. (**a**) Nine acidic amino acid residues with the highest SASA in TTR. 1–9 represent acidic amino acid residues of TTR in descending order of SASA. (**b**) The position of nine acidic amino acid residues with the highest SASA in TTR (marked in red). (**c**) Tryptophan fluorescence. (**d**) ANS fluorescence. (**e**) CD spectra. (**f**) Secondary structure content. The concentrations of the four proteins were 10 μM.

For mutagenesis, we chose acidic amino acid residues with higher solvent-accessible surface area (SASA) calculated using Pymol software (Version 3.0.4) [33,34]. As shown in Figure 1a, there are nine residues with higher SASA, including 62E, 51E, 61E, 63E, 38D, 66E, 39D, 99D, and 42E. Furthermore, the schematic structure of the TTR protein in Figure 1b demonstrates that all nine amino acid residues are exposed on the surface of the protein. Therefore, the mutation sites were selected from the above nine amino acid residues. All mutants were expressed in *Escherichia coli* (*E. coli*) BL21 using pCold II as the gene expression vector and purified by affinity chromatography.

The influence of the mutated amino acid type on the inhibitory effect of recombinant TTR was first explored. Five mutation sites were selected, and the sets of mutation and naming methods are listed in Table 1. The expression of the different recombinant TTRs was analyzed by sodium dodecyl sulfate-polyacrylamide gel electrophoresis (SDS-PAGE). As can be seen in Figure S1, there are clear protein bands near 15 KDa and weak bands near 35 kDa in all lanes, corresponding to monomers and dimers, respectively, indicating the successful expression and purification of m1/2TTR-5K/5R/5H. It has been demonstrated that both monomeric and dimeric TTR can interact with Aβ and inhibit Aβ aggregation [35]. Therefore, proteins with a mixture of species (monomers and dimers) were utilized in the following research. The effects of m1/2TTR-5K/5R/5H on the inhibition of Aβ_40_ fibrillization were explored by in situ thioflavin T (ThT) fluorescence experiments. As illustrated in Figure S2, the kinetics of Aβ_40_ aggregation showed a sigmoidal curve, indicating that Aβ_40_ alone aggregated into fibrils. Compared with TTR, m1/2TTR-5K/5R/5H all showed enhanced inhibitory potency of Aβ_40_ aggregation. This may be because the mutation of acidic amino acids in TTR to basic amino acids increased the positive charge on its surface and enhanced the electrostatic interactions with Aβ_40_, thus leading to the improved inhibitory effect [36,37]. Among them, the R mutants (m1/2-TTR-5R) showed the strongest inhibitory potency; m1TTR-5R and m2TTR-5R at 1 μM inhibited ThT fluorescence by 66% and 72%, respectively, higher than TTR (35%), m1TTR-5K (45%), m1TTR-5H (40%), m2TTR-5K (57%), and m2TTR-5H (61%) (Figure S3). R has a higher isoelectric point than K and H, so the m1/2TTR-5R surface carries more positive charges, making it present stronger electrostatic interactions with Aβ_40_. In addition, at 1 μM, the inhibitory effect of m2TTR-5K/5R/5H on Aβ_40_ aggregation was more potent than that of m1TTR-5K/5R/5H. This may be due to the extensive electrostatic interactions provided by m2TTR-5K/5R/5H. The lag times of Aβ_40_ treated with m1/2TTR-5K/5R/5H are listed in Table S1. Both m1TTR-5R and m2TTR-5R could prolong the lag time of Aβ_40_ (Figure S2b,e), extending the lag time from 9.1 ± 2.4 h to 85.5 ± 1.5 h and 118.8 ± 3.0 h, respectively, at 4 μM (Table S1), indicating that the nucleation of Aβ_40_ was inhibited. The above results prove that the R mutants possessed the most potent inhibitory effect. Therefore, the R mutants (simplified as TTR-nR) were taken as a representative in the subsequent experiments to explore the enhanced inhibitory potency of recombinant TTR targeting Aβ_40_ aggregation and the mechanism, and the effect of the number of mutation sites on the inhibitory capability was examined. The sets of mutation and naming schemes are shown in Table 2. The m2TTR-5R was selected as a control and simply denoted as TTR-5R. More detailed characterization is as follows.

The molecular weights of TTR-5R/7R/9R were determined by SDS-PAGE and matrix-assisted laser desorption/ionization time-of-flight mass spectrometry (MALDI-TOF MS). The purified proteins (TTR-7R and TTR-9R) were composed of monomers (~15 kDa) and dimers (~35 kDa) (Figure S4), in agreement with the previously reported literature [38,39]. Among them, the content of TTR-9R was low and mainly present as a dimer. MALDI-TOF MS exhibited that the molecular weights of TTR, TTR-5R, TTR-7R, and TTR-9R were 15.2, 15.3, 15.4, and 15.6 kDa, respectively, which were consistent with the theoretical values (Figure S5a) [39,40]. The sizes of TTR and TTR-5R/7R/9R were measured by dynamic light scattering. As shown in Figure S5b, the hydrodynamic diameter (Dh) of TTR was 5.6 nm, which was in good agreement with previous reports [20]. In contrast, the Dh values of TTR-5R, TTR-7R, and TTR-9R increased to 7.5, 8.7, and 142.0 nm, respectively. These results indicate that TTR-9R was prone to dimer formation, unstable in solution, and easy to aggregate. Under physiological conditions (pH 7.4), TTR was almost electrically neutral with a ζ-potential value of 0.17 mV, while the ζ-potential of TTR-5R/7R/9R were all higher than TTR, and the ζ-potential was gradually increased with the increase in the number of mutation sites, with potential values of 10.2, 13.5, and 16.1 mV, respectively (Figure S5c). The result suggests that the increase in the number of mutation sites improved the positive charge of TTR-nR, which may be beneficial in enhancing the inhibitory potency.

However, an increase in the number of mutation sites may cause changes in the structure of TTR-nR, thereby affecting their properties. The effect of increasing the number of mutation sites on TTR was evaluated using tryptophan fluorescence spectra [41,42]. As shown in Figure 1c, the fluorescence intensity of TTR-9R at 343 nm decreased significantly compared with TTR, TTR-5R, and TTR-7R, indicating that the microenvironment near Trp41 of TTR-9R was significantly altered [38]. Moreover, the hydrophobicity of the proteins was investigated using an 8-anilino-1-naphthalenesulfonic acid (ANS) fluorescence probe. Upon the addition of TTR and TTR-5R/7R/9R, the maximum emission of ANS was blue-shifted from 535 nm to 475 nm, which was accompanied by an increase in fluorescence intensity. The hydrophobicity of TTR-5R and TTR-7R was almost the same as that of TTR, whereas the fluorescence intensity of TTR-9R was significantly reduced (Figure 1d), indicating that the hydrophobicity of TTR-9R was weakened. The conformational information was characterized by circular dichroism (CD) spectroscopy (Figure 1e) and quantitatively analyzed in Figure 1f. The β-sheet, α-helix, and turn contents of TTR were 29.1%, 18.0%, and 10.1%, respectively, which was consistent with the known crystal structure of TTR [39,43], and those of TTR-9R were 23.8%, 16.2%, and 12.5%, respectively. Compared with TTR-5R and TTR-7R, the secondary structure content of TTR-9R was significantly altered due to the increase in the mutation sites.

### 2.2. Enhanced Inhibitory Potency of TTR-nR

**Figure 2:**
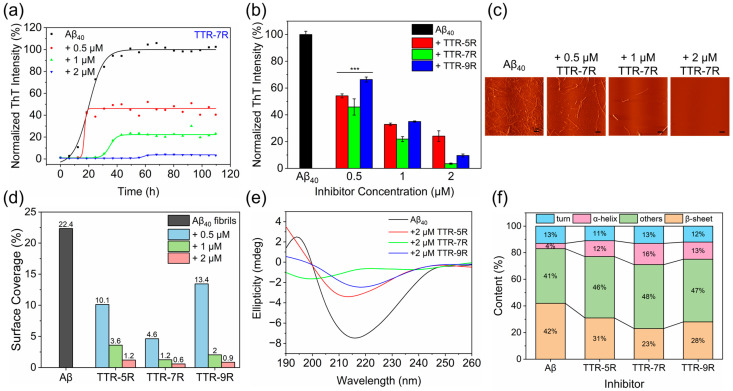
Inhibition of Aβ_40_ fibrillization by TTR-7R. (**a**) ThT fluorescence kinetic assay of Aβ_40_ monomers incubated with TTR-7R. (**b**) Normalized ThT fluorescence intensity of Aβ_40_ incubated with different inhibitors for 100 h. *** *p* < 0.001, compared to the control group. (**c**) AFM images of Aβ_40_ incubated with different concentrations of TTR-7R for 100 h. Scale bar, 1 μm. (**d**) Surface coverage of Aβ_40_ incubated without or with various agents (0.5–2 μM) for 100 h. (**e**) Far-UV circular dichroism spectra of Aβ_40_ incubated with different agents (2 μM) at 100 h. (**f**) Secondary structure content of Aβ_40_ alone or with different inhibitors. The inhibitor concentration was 2 μM. The Aβ_40_ concentration was 25 μM in all experiments.

The effects of TTR-5R/7R/9R on Aβ_40_ fibrillization were investigated by ThT kinetic experiments. As mentioned earlier, the mutant form is a kinetically stable monomer. Under the experimental conditions (pH 7.4), the ThT fluorescence of TTR and TTR-5R/7R/9R alone did not change significantly with the extension of incubation time, indicating that TTR and TTR-5R/7R/9R did not readily aggregate into fibrils (Figure S6). As illustrated in Figure 2a and Figure S7, the kinetics of Aβ_40_ alone showed a typical sigmoidal curve. TTR-5R/7R/9R all effectively inhibited Aβ_40_ aggregation in a concentration-dependent manner, and the inhibitory capability was stronger than that of TTR. Among them, TTR-7R showed the best inhibitory potency, which could inhibit 54% of ThT fluorescence at 0.5 μM, while TTR-5R and TTR-9R inhibited only 46% and 34%, respectively (Figure 2b). In particular, at the concentration of 2 μM, TTR-7R could almost completely inhibit Aβ_40_ aggregation, which was significantly stronger than that of previously reported alkalized TTR (TTR-B3) [44] and alkalized bovine serum albumin (BSA-B4) [13]. The lag times of different protein inhibitors were calculated and are listed in Table S2. The lag time of Aβ_40_ alone was 9.4 ± 0.3 h. TTR-7R at 0.5 μM prolonged the lag time to 15.5 ± 0.2 h, and the lag time of Aβ_40_ gradually increased with increasing protein concentration, indicating that TTR-7R could inhibit the nucleation of Aβ_40_. The above results indicate an enhanced inhibitory effect of TTR-5R/7R/9R compared with TTR, with TTR-7R exhibiting the strongest inhibitory capacity and inhibiting Aβ_40_ nucleation even at low concentrations. Notably, inhibition of Aβ fibril formation may also prevent the toxicity of monomeric TTR, as well as slow the progression of Aβ pathology [28].

The effect of TTR-5R/7R/9R on the morphology of Aβ_40_ aggregates was investigated by atomic force microscopy (AFM). As shown in Figure 2c, Aβ_40_ alone aggregated into long and dense fibrils. Upon the addition of TTR-5R/7R/9R, the fibrils became sparse, and only broken short fibrils were observed (Figure 2c and Figure S8). Of note, the number of Aβ_40_ fibrils treated with TTR-7R was the lowest at all concentrations, with relatively short dimensions. In particular, after treatment with 2 μM of TTR-7R, the fibrils almost disappeared, with only a few small particles observed (Figure 2c). For quantitative analysis of the AFM images, the surface coverage of Aβ_40_ fibrils with different protein inhibitors was estimated by Image J (Version 1.54f, free software: https://imagej.net) and is summarized in Figure 2d. The surface coverage of Aβ_40_ fibrils alone was 22.4%, and it decreased to 4.6% with 0.5 μM TTR-7R and to 0.6% with 2 μM, indicating the potent inhibitory capability of TTR-7R. The above results indicate that TTR-7R could significantly inhibit Aβ_40_ aggregation and reduce the production of Aβ_40_ fibrils compared with TTR-5R and TTR-9R.

To comprehend the conformational changes in Aβ_40_ without or with different protein inhibitors, CD measurements were performed. As shown in Figure 2e, after 100 h of incubation, Aβ_40_ alone showed a maximum positive peak near 195 nm and a maximum negative peak near 216 nm, indicating the formation of β-sheet-rich structures. After co-culture with 2 μM TTR-5R/7R/9R, the characteristic peaks of β-sheet structure were decreased, proving that TTR-5R/7R/9R could inhibit the formation of β-sheet-rich structure. Especially when treated with 2 μM TTR-7R, the typical characteristic peaks of β-sheet structure did not appear, proving that the on-pathway aggregation of Aβ_40_ was altered. To further quantify the influence of TTR-5R/7R/9R on Aβ_40_ conformation, the secondary structure content was calculated using the BeStSeL algorithm (Figure 2f). TTR-7R (2 μM) reduced the β-sheet content from 42% to 23%, while TTR-5R and TTR-9R reduced the β-sheet content to 31% and 28%, respectively. These results indicate that TTR-7R could effectively block the transformation of the secondary structure of Aβ_40_ into the β-sheet-rich conformation, which was stronger than that of TTR-5R and TTR-9R.

Taken together, these results suggest that the increase in basic amino acids enhanced the inhibition of Aβ_40_ aggregation by TTR-5R/7R/9R relative to TTR, which was attributed to the increase in protein isoelectric point, resulting in an increase in the surface positive charge and enhanced electrostatic interactions with Aβ_40_. However, TTR-9R had a weaker inhibitory capacity than TTR-7R, which may be due to the structural changes in TTR-9R caused by the increase in mutation sites, thus reducing the hydrophobic interactions with Aβ_40_. This will be discussed in more detail next.

### 2.3. Mechanistic Analysis of TTR-5R/7R/9R Modulating Aβ Fibrillization

**Figure 3:**
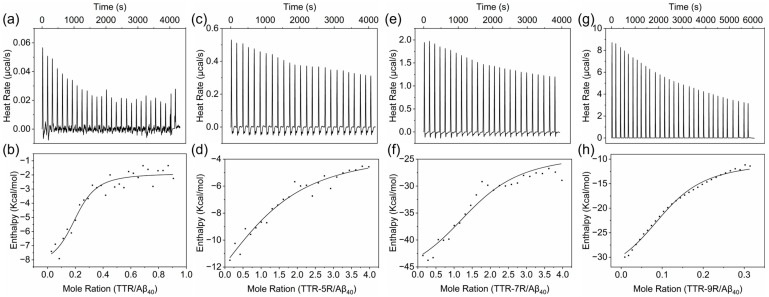
Calorimetric data for titration of (**a**,**b**) TTR, (**c**,**d**) TTR-5R, (**e**,**f**) TTR-7R, and (**g**,**h**) TTR-9R into Aβ_40_ (20 μM) at 25 °C. The concentrations of TTR and TTR-5R/7R/9R were all 40 μM.

To elucidate the inhibitory mechanism, the affinity between TTR-5R/7R/9R and Aβ_40_ was determined by isothermal titration calorimetry (ITC) assay. Thermodynamic parameters, such as enthalpy (Δ*H*), entropy (Δ*S*), Gibbs free energy (Δ*G*), and association constant (*K*a), were calculated by fitting the titration data using an independent site model (Figure 3). As shown in Table 3, the *K*a of TTR, TTR-5R, TTR-7R, and TTR-9R were 1.55, 2.07, 17.00, and 1.26 × 10^4^ M^−1^, respectively. The larger value of *K*a indicated a higher binding affinity of proteins. Compared with TTR, TTR-7R showed a significantly higher affinity with Aβ_40_ and was also higher than TTR-5R and TTR-9R. It is known that hydrogen bonding and/or electrostatic interactions can cause negative enthalpy changes, while hydrophobic interactions can lead to positive entropy changes [45]. The negative Δ*H* value indicated that the binding events mainly contributed to electrostatic interactions. The changes in *T*Δ*S* value demonstrated that TTR-5R and TTR-7R largely retained the hydrophobic interactions with Aβ_40_, while TTR-9R had weaker hydrophobic interactions with Aβ_40_ due to the reduction in exposed hydrophobic sites. Furthermore, by comparing the *T*Δ*S* and Δ*H* values of TTR-5R/7R/9R and Aβ_40_, it can be speculated that the difference in *K*a values is mainly attributed to the different electrostatic interactions.

**Figure 4:**
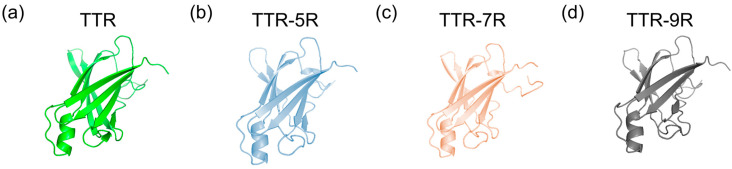
Structures of (**a**) TTR monomer (PDB ID: 1DVQ), (**b**) TTR-5R monomer, (**c**) TTR-7R monomer, and (**d**) TTR-9R monomer.

To further characterize the conformational information of TTR-5R/7R/9R, the 3D model of TTR-5R/7R/9R was reconstructed using AlphaFold2 [46,47]. As revealed by Figure 4, TTR-5R/7R/9R showed similar structures to TTR, but the β-sheet content was TTR-5R ≈ TTR-7R > TTR-9R (Figure 1f and Figure 4b–d). The difference in β-sheet structure was mainly caused by the different number of mutation sites. TTR-5R and TTR-7R did not significantly change the structure of TTR, but the structure of TTR-9R was altered, which may lead to its reduced hydrophobicity and stability, as confirmed by the characterization results (Figure 1 and Figure S5). In summary, we considered that hydrophobic interactions worked in stabilizing the TTR-7R-Aβ complex, and enhanced electrostatic interactions further reinforced the interactions. Therefore, TTR-7R bound Aβ via hydrophobic interactions and enhanced electrostatic interactions, thus showing the strongest inhibitory effect.

### 2.4. Attenuation of Aβ_40_-Induced Cytotoxicity

**Figure 5:**
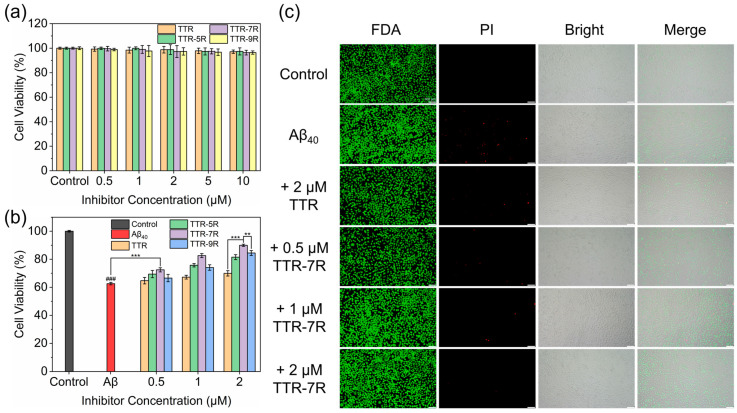
SH-SY5Y cell assays with various agents. (**a**) Viability of SH-SY5Y cells incubated with TTR and TTR-5R/7R/9R. (**b**) Detoxification of Aβ_40_-induced SH-SY5Y cytotoxicity. The viability of cells treated with PBS buffer only was defined as 100%. The data are presented as mean ± SD (*n* = 6). ### *p* < 0.001, compared to the control group. Values of *p* < 0.01 and *p* < 0.001 for the pairs of data sets are marked with ** and ***, respectively. (**c**) Observation of the protection of TTR-7R on SH-SY5Y cells by FDA/PI double staining. FDA-positive cells present viable cells, while PI-positive cells present dead cells. Scale bar, 100 μm. The aged Aβ_40_ concentration was 25 μM.

The biocompatibility and detoxification capability of TTR-5R/7R/9R on the Aβ_40_-induced cytotoxicity was assessed by the 3-(4,5-dimethylthiazol-2-yl)-2,5-diphenyltetrazolium bromide (MTT) assay with SH-SY5Y cells. The cell viability assays illustrated that TTR and TTR-5R/7R/9R exhibited inconspicuous cytotoxicity even at a high concentration of 10 μM, indicating their good biocompatibility for biological applications (Figure 5a). As illustrated in Figure 5b, pre-incubated Aβ_40_ species significantly reduced the cell viability to 62%, confirming the neurotoxicity of Aβ_40_. TTR slightly rescued the cell viability to 70% at 2 μM, demonstrating its moderate detoxification. In contrast, TTR-7R significantly restored cell viability and improved the cell viability to 90% at 2 μM, which was more potent than those of TTR-5R and TTR-9R, highlighting the capability of TTR-7R to protect neurons from Aβ_40_-induced neurotoxicity at relatively low concentrations.

The function of TTR-7R in protecting the SH-SY5Y cells from the damage of toxic Aβ_40_ was further verified by fluorescein diacetate/propidium iodide (FDA/PI) double staining assays. In Figure 5c, the cells alone presented only green fluorescence. The Aβ_40_ group evidently exhibited more PI-positive staining, indicating the death of cells. After incubation with TTR, the number of PI-positive cells decreased slightly. Remarkably, the dead cells almost disappeared with TTR-7R at 2 μM. The results demonstrate the effective detoxification effect and good biocompatibility of TTR-7R, which was more potent than TTR, TTR-5R, and TTR-9R.

### 2.5. In Vivo Caenorhabditis elegans (C. elegans) Assays

**Figure 6:**
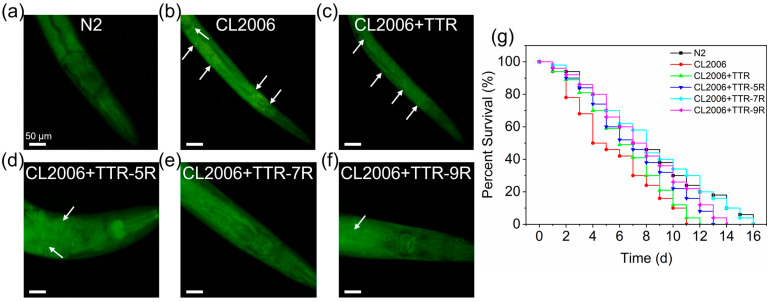
In vivo assays with nematodes. Representative fluorescence images of Aβ deposits in (**a**) N2 and (**b**) CL2006 nematodes co-stained with ThT. Fluorescence images of inhibiting Aβ deposits in CL2006 nematodes with (**c**) TTR, (**d**) TTR-5R, (**e**) TTR-7R, or (**f**) TTR-9R. The white arrow in the figures represents Aβ deposits in *C. elegans*. Scale bar, 50 μm. (**g**) Survival curves of N2 or CL2006 strains after co-incubation with TTR and TTR-5R/7R/9R. The concentrations of TTR and TTR-5R/7R/9R were all 2 μM.

As *C. elegans* mutant CL2006 expresses Aβ_42_ in the body wall muscle [48,49], *C. elegans* was employed as an in vivo model to test the therapeutic effect of TTR-5R/7R/9R. As a control, wild-type N2 nematodes did not express Aβ_42_, and there was no amyloid plaque formation (Figure 6a). As shown in Figure 6b, a large number of green fluorescent spots were observed in CL2006, indicating the accumulation and deposition of Aβ. There were still significant fluorescent plaques in CL2006 nematodes treated with 2 μM of TTR, suggesting that the scavenging effect of TTR on Aβ plaque deposition was weak at this concentration (Figure 6c). The scavenging capacity of TTR-7R was remarkably enhanced, and no obvious Aβ deposits were present in CL2006 nematodes administered with 2 μM of TTR-7R (Figure 6e). As a control, some plaques were observed in CL2006 nematodes treated with 2 μM of TTR-5R and TTR-9R (Figure 6d,f). The results indicate that, compared with TTR, TTR-5R/7R/9R could effectively scavenge Aβ_42_ deposits in CL2006 nematodes, and TTR-7R demonstrated a more potent scavenging capability.

The benefits of TTR and TTR-5R/7R/9R on the lifespan of *C. elegans* were then investigated. As depicted in Figure 6g, the lifespan of N2 nematodes was 16 d, while CL2006 nematodes without any treatment were all paralyzed and died within 11 d. TTR had little effect on the lifespan of CL2006 nematodes. However, TTR-7R postponed the death time of CL2006 nematodes from 11 to 16 d. In contrast, TTR-5R and TTR-9R only prolonged nematode lifespan to 13 and 14 d, respectively. The above results prove that TTR-5R/7R/9R could mitigate the toxicity more efficiently than TTR, among which TTR-7R could act as a potent scavenger to eliminate amyloid deposition in vivo, further demonstrating the potential of TTR-7R for AD treatment.

## 3. Materials and Methods

### 3.1. Materials

Ampicillin (Amp), Isopropyl β-d-Thiogalactoside (IPTG), Coomassie Brilliant Blue R-250, 1,1,1,3,3,3-hexafluoro-2-propanol (HFIP), thioflavin T (ThT), 3-(4,5-dimethylthiazol-2-yl)-2,5-diphenyltetrazolium bromide (MTT), and dimethyl sulfoxide (DMSO) were purchased from Sigma-Aldrich (St. Louis, MO, USA). Fluorescein diacetate (FDA) and propidium iodide (PI) were received from DingGuo Biotech (Beijing, China). Fluorodeoxyuridine (FUDR) and 1-Anilinonaphthalene-8-sulfonic (ANS) were obtained from Shanghai Aladdin Bio-Chem Technology (Shanghai, China). Aβ_40_ (>95%, synthetic powder) was from GL Biochem (Shanghai, China). Human neuroblastoma cells (SH-SY5Y) were provided by the Cell Bank of the Chinese Academy of Sciences (Shanghai, China). DMEM/F12 medium, fetal bovine serum (FBS), trypsin, and penicillin–streptomycin solution were purchased from Gibco (New York, NY, USA). The wild-type N2 and transgenic CL2006 strains of *Caenorhabditis elegans* (*C. elegans*) were from the Caenorhabditis Genetics Center (University of Minnesota, Minneapolis, MN, USA). *Escherichia coli* (*E. coli*) OP50 was acquired from Henan University (Kaifeng, China). Other reagents were of the highest purity and obtained from local sources. All chemical solutions were prepared using ultrapure water.

### 3.2. Screening of Mutation Sites

Based on solvent-accessible surface area (SASA) values, amino acid residues of proteins can be classified as buried or exposed [33]. The SASA of acidic amino acids in TTR was calculated using Pymol software (Version 3.0.4), and the residues with higher SASA were selected for mutation. The specific mutation schemes are shown in Table 1 and Table 2.

### 3.3. Expression and Purification of Recombinant TTR

TTR, m1/2TTR-5K/5R/5H, and TTR-7R/9R were expressed in *E. coli* (BL21) and purified as previously described [20]. Briefly, pCold II-TTR, m1/2TTR-5K/5R/5H, and TTR-7R/9R expression vectors were designed based on the amino acid sequences of the proteins. Then, vectors were transformed into *E. coli* BL21. The BL21 seed solution was inoculated into an LB medium containing 100 mg/L ampicillin at 37 °C for 12 h. When the OD600 value reached 0.8–1.0, 1 mM of IPTG was added and cultured for another 4–6 h (37 °C, 220 rpm). Next, the cells were collected by centrifugation at 4 °C (4000 r/min, 30 min), and the cell pellet was disrupted by sonication in lysis buffer (20 mM Tris-HCl, pH 7.4, 150 mM NaCl, 20 mM imidazole, 1 mM DTT). After 30 min of disruption, the cells in the supernatant were collected by centrifugation at 10,000 r/min (30 min, 4 °C) and filtered. Subsequently, the solutions were purified by an affinity chromatography column filled with Ni-NTA Sepharose resin (GE Healthcare, Uppsala, Sweden). The column was washed with lysis buffer and finally eluted with elution buffer (20 mM Tris-HCl, 500 mM NaCl, 500 mM imidazole, pH 7.5) to recover the purified protein. Before use, the protein solutions were dialyzed at 4 °C for 3 d, lyophilized, and stored at −20 °C.

### 3.4. Sodium Dodecyl Sulfate-Polyacrylamide Gel Electrophoresis (SDS-PAGE) Analysis of Various Proteins

To determine the purity of TTR, m1/2TTR-5K/5R/5H, and TTR-7R/9R, the SDS-PAGE assay was carried out. The diluted sample (20 μL) was incubated with a loading buffer in boiling water for 15 min and analyzed by SDS-PAGE at 80 mV. Then, TTR, m1/2TTR-5K/5R/5H, and TTR-7R/9R were stained by Coomassie Brilliant Blue R-250 for 15 min, and the target proteins were quantified by grayscale analysis after decoloration.

### 3.5. Aβ_40_ Monomer Solution Preparation

The lyophilized Aβ_40_ monomers were dissolved in HFIP (1.0 mg/mL) and set quietly at 4 °C for 2 h, followed by sonicated in an ice bath for 20 min. Then, the supernatant was collected and lyophilized with a vacuum freeze dryer (Labconco, Kansas City, MO, USA). Before use, the processed Aβ_40_ monomers were diluted to 275 µM in NaOH solution (20 mM). In inhibiting Aβ_40_ fibrillization experiments, the Aβ_40_ solution (275 µM) was diluted with PBS buffer containing various inhibitors to the final Aβ_40_ concentration of 25 µM.

### 3.6. ThT Fluorescence Assay

ThT dyes were used to monitor the effects of different inhibitors on the kinetics of Aβ_40_ aggregation. Briefly, the pretreated Aβ_40_ (25 μM) was co-incubated with different inhibitors in a microplate reader (TECAN Infinite, Salzburg, Austria) at 37 °C. The ThT fluorescence intensity was recorded (excitation/emission, 440/480 nm). The final fluorescence results were obtained by subtracting the fluorescence intensity of the Aβ_40_-free solution. All kinetic curves were the independent average of triplicates. The kinetic curves were fitted using the following equation:

(1) $$y=y_{0}+\frac{y_{\max}-y_{0}}{1+{\;e}^{-(t\;-{\;t}_{1/2})k}}$$

where y_0_ and y_max_ are the initial and maximum fluorescence intensity, respectively; t_1/2_ is the time when the intensity reaches half of the maximum fluorescence value; and k is the first-order kinetic constant. The lag time (T_lag_) was then calculated according to the following equation [50]:

(2) $$T_{\mathrm{lag}}=t_{1/2}-\;\frac{2}{k}$$

### 3.7. Characterization of TTR and TTR-5R/7R/9R

The molecular weights were determined by matrix-assisted laser desorption/ionization time-of-flight mass spectrometry (MALDI-TOF MS) (Autoflex Tof/TofIII, Bruker Daltonics, Billerica, MA, USA). The size distribution and ζ-potential were detected using a Zetasizer Nano (Malvern Instruments, Worcestershire, UK). The tryptophan fluorescence spectra of TTR and TTR-5R/7R/9R (0.1 mg/mL) were measured with a fluorescence spectrometer (PerkinElmer LS-55, Waltham, MA, USA) in PBS buffer. The hydrophobicity was characterized by ANS fluorescence spectra. The structure information of TTR and TTR-5R/7R/9R was analyzed using a circular dichroism (CD) spectrometer (J-810, Jasco, Tokyo, Japan). The secondary structure content was obtained by analysis using the BeStSeL web server (https://bestsel.elte.hu).

### 3.8. Atomic Force Microscopy (AFM) Analysis

The sample (50 μL) was deposited on a freshly cleaved mica substrate for 5 min. Then, the substrates were washed with ultrapure water 5 times and finally dried overnight. The morphology of Aβ_40_ fibrils was observed using a multimode AFM (CSPM5500, Benyuan, Beijing, China) under tapping mode. Three different areas per sample were selected for observation to ensure consistency.

### 3.9. CD Spectroscopy

The CD spectra of samples were monitored with a 0.1 cm path-length quartz cell using the CD spectrometer mentioned above at room temperature. The spectral range was 190–260 nm with a bandwidth of 1 nm at a scanning speed of 200 nm/min. The data were the average of three scans and subtracted against the background of samples that did not contain Aβ_40_.

### 3.10. Isothermal Titration Calorimetry (ITC) Experiment

The binding affinity of TTR and TTR-5R/7R/9R to Aβ_40_ was measured by an Affinity ITC (TA Instruments, New Castle, DE, USA). The freshly prepared Aβ_40_ (20 μM) was dissolved in a PBS buffer and added to the sample pool. TTR or TTR-5R/7R/9R solution (40 μM) was consecutively titrated into Aβ_40_ solution every 300 s at 25 °C. The dilution heat was obtained as the background by titrating proteins into the buffer. All samples were degassed in a vacuum for 10 min. Data were fitted using Launch Nano analyze software (version 3.11.0, TA Instruments). The independent site model was used for fitting to determine the corresponding association constant (*K*a), the enthalpy change (Δ*H*), and the entropy change (Δ*S*).

### 3.11. Cell Viability Assay

MTT assays were conducted to understand the effects of TTR-5R/7R/9R on Aβ_40_-induced cytotoxicity. SH-SY5Y cells were cultured in DMEM/F12 medium containing 10% FBS and 1% penicillin–streptomycin in a cell incubator (37 °C and 5% CO_2_). The SH-SY5Y cells (8 × 10^3^ cells/well, 80 µL) were added to a sterile 96-well plate and incubated for 24 h. Then, the pre-prepared Aβ_40_ samples containing different inhibitors (20 µL) were added. After 24 h of incubation, 10 µL MTT (5.5 mg/mL) were added to each well, followed by cultured for 4 h. The supernatant was replaced with 100 μL DMSO to dissolve the formazan crystals. After shaking at 37 °C for 20 min, the absorbance at 570 nm was recorded with a plate reader. All of the data were repeated six times. The experimental results were obtained with respect to the control untreated cells. The cell viability was calculated using the following equation:

(3) $$\mathrm{Cell}\;\mathrm{viability}\left(\%\right)=\frac{100\%\;\times \;({\mathrm{OD}}_{\mathrm{Treated}}-{\mathrm{OD}}_{\mathrm{Background}})}{{\mathrm{OD}}_{\mathrm{Control}}-{\mathrm{OD}}_{\mathrm{Background}}}$$

where OD_Treated_ was obtained in the presence of protein inhibitors.

### 3.12. FDA/PI Double Staining Assay

FDA/PI double staining assays were performed to identify the dead and live cells. SH-SY5Y cells were pretreated with different protein inhibitors, as described in the Section 3.11. After being cultured, the cell culture media were replaced with FDA/PI working solutions (10 μg/mL FDA and 5 μg/mL PI) and kept in the dark at 37 °C for 15 min. Before imaging, the stained cells were washed with PBS buffer 5 times. All images were captured under an inverted fluorescence microscope (TE2000-U, Nikon, Tokyo, Japan).

### 3.13. C. elegans In Vivo Experiment

*C. elegans* wild-type N2 and mutant CL2006 were cultured on a nematode growth medium (NGM) (3 g/L NaCl, 1 mM CaCl_2_, 1 mM MgSO_4_, 5 μg/mL cholesterol, 17 g/L agar, 2.5 g/L peptone, 250 mM KH_2_PO_4_) containing *E. coli* OP50 at 20 °C.

To verify the scavenging capability of TTR or TTR-5R/7R/9R on amyloid deposits in CL2006 nematodes, the protein inhibitors (2 μM, 200 μL) were uniformly spread onto the NGM medium containing N2 or CL2006 *C. elegans* at L4 larval stage, and incubated at 20 °C for 3 d. After fixation with 4% paraformaldehyde at 4 °C for 24 h, the nematodes were stained with 10 µM ThT for an additional 4 h. The stained nematodes were observed with the inverted fluorescence microscope mentioned above.

In the lifespan assays, 60 N2 or CL2006 nematodes were transferred to fresh NGM plates containing TTR or TTR-5R/7R/9R (2 µM, 200 µL), respectively, and FUDR (150 µM, 300 µL) was added to inhibit the nematodes spawning. The number of surviving nematodes was recorded every day until all nematodes were completely dead. Each group of survivors was transferred to a new NGM plate containing *E. coli* OP50 and corresponding agents every 3 d. The nematodes were scored as dead when they did not respond to mechanical stimulation with a platinum wire.

### 3.14. Statistical Analysis

All data are presented as mean values ± standard deviation (SD). All statistical analyses were conducted using one-way analysis of variance (ANOVA) followed by a statistical comparison with a Tukey test. *p* < 0.05 or less was considered statistically significant.

## 4. Conclusions

In this work, various recombinant TTRs were developed by cationic surface charge engineering, and the effects of the mutated amino acid type (m1/2TTR-5K/5R/5H) and the number of mutation sites (TTR-7R/9R) on the inhibition of Aβ aggregation were investigated. A series of experimental studies showed that the inhibitory capacity of all recombinant TTRs was enhanced compared with TTR. Among them, the TTR mutated to arginine (TTR-nR) had a stronger inhibitory effect. Of note, as the number of mutation sites increased (TTR-5R, TTR-7R, and TTR-9R), TTR-7R exhibited the most potent Aβ inhibitory activity. TTR-7R could effectively modulate the on-pathway Aβ aggregation, reduce Aβ_40_-induced cytotoxicity from 38% to 11%, promote the clearance of Aβ deposition, and prolong the lifespan of CL2006 worms by 5 d at a very low concentration (2 μM). Mechanistic analysis revealed that this was attributed to retained hydrophobic interactions and enhanced electrostatic interactions between TTR-7R and Aβ species. The research has presented important insights into the development of recombinant protein inhibitors for the treatment of neurodegenerative diseases.

## Figures and Tables

**Table 1:** TTR mutation scheme with five site mutations.

Mutant	Mutation Site
m1TTR-5K ^1^	D38K, E51K, E61K, E62K, E63K
m1TTR-5R	D38R, E51R, E61R, E62R, E63R
m1TTR-5H	D38H, E51H, E61H, E62H, E63H
m2TTR-5K ^2^	D38K, E51K, E62K, E66K, D99K
m2TTR-5R	D38R, E51R, E62R, E66R, D99R
m2TTR-5H	D38H, E51H, E62H, E66H, D99H

^1^ m1TTR-5K/5R/5H: Five acidic amino acid residues with the highest SASA in TTR were selected as mutation sites. The m1 represents the first type of mutant. ^2^ m2TTR-5K/5R/5H: Five discrete acidic amino acid residues with higher SASA in TTR were selected as mutation sites. The m2 represents the second type of mutant.

**Table 2:** Mutation schemes for TTR-7R and TTR-9R.

Mutant ^1^	Mutation Site
TTR-7R	D38R, E51R, E61R, E62R, E63R, E66R, D99R
TTR-9R	D38R, D39R, E42R, E51R, E61R, E62R, E63R, E66R, D99R

^1^ TTR-7R and TTR-9R are mutations of 7 and 9 acidic amino acids in TTR to arginine R, respectively.

**Table 3:** Thermodynamic parameters and association constants obtained by fitting ITC data.

	TTR	TTR-5R	TTR-7R	TTR-9R
*K*a (×10^4^ M^−1^)	1.55 ± 0.14	2.07 ± 1.10	17.00 ± 2.10	1.26 ± 0.77
Δ*H* (Kcal/mol)	−6.66 ± 3.90	−7.53 ± 1.56	−9.06 ± 1.67	−23.90 ± 1.36
*T*Δ*S* (Kcal/mol)	1.79	−1.84	−1.93	−4.37
Δ*G* (Kcal/mol)	−8.45	−5.69	−7.13	−19.53

## Data Availability

The data that support the findings of this study are available from the corresponding author upon reasonable request.

## Supplementary Materials

The following supporting information can be downloaded at https://www.mdpi.com/article/10.3390/molecules29215023/s1, Table S1: Lag times (*T*_lag_) of Aβ_40_ (25 μM) aggregation kinetics in the presence of m1/2TTR-5K/5R/5H (4 μM); Table S2: *T*_lag_ for amyloid formation kinetics of Aβ_40_ (25 μM) incubated with different agents; Figure S1: SDS-PAGE analysis of TTR mutated at five sites; Figure S2: Effects of m1TTR-5K, m1TTR-5R, m1TTR-5H, m2TTR-5K, m2TTR-5R, and m2TTR-5H on Aβ_40_ aggregation measured by ThT fluorescence assay; Figure S3: Final ThT fluorescence of Aβ_40_ (25 μM) incubated with different inhibitors for 120 h; Figure S4: SDS-PAGE bands of TTR-7R and TTR-9R; Figure S5: MALDI-TOF MS, size distribution, and ζ-potential of TTR and TTR-5R/7R/9R; Figure S6: Time-dependent ThT fluorescence changes of TTR, TTR-5R, TTR-7R, and TTR-9R alone; Figure S7: ThT fluorescence kinetic assay of Aβ_40_ incubated with TTR, TTR-5R, and TTR-9R; Figure S8: AFM images of Aβ_40_ (25 μM) aggregates alone or co-incubated with different concentrations of TTR-5R and TTR-9R.
