# Supplementary material for: Cationic Surface Charge Engineering of Recombinant Transthyretin Remarkably Increases the Inhibitory Potency Against Amyloid β-Protein Fibrillogenesis
Source: Molecules. 2024 Oct 24;29(21):5023. doi: 10.3390/molecules29215023 (PMC11547489; doi:10.3390/molecules29215023)
Supplement: Supplementary file 1 [file molecules-29-05023-s001.zip › molecules-3245603-supplementary.pdf]

Supporting information

Cationic Surface Charge Engineering of Recombinant Transthyretin Remarkably Increases the Inhibitory Potency Against Amyloid β-Protein Fibrillogenesis

Xiaoding Lin †, Ting Xu †, Wenqi Hou, Xiaoyan Dong and Yan Sun *

Key Laboratory of Systems Bioengineering and Frontiers Science Center for Synthetic Biology (Ministry of Education), Department of Biochemical Engineering, School of Chemical Engineering and Technology, Tianjin University, Tianjin 300350, China; linxiaoding@tju.edu.cn (X.L.); xuting77@tju.edu.cn (T.X.); hwq@tju.edu.cn (W.H.); d_xy@tju.edu.cn (X.D.)

* Correspondence: ysun@tju.edu.cn; Tel./Fax: +86-22-27403389

† These authors contributed equally to this paper.

Table S1. Lag times (T_{lag}) of $A\beta_{40}$ (25 μM) aggregation kinetics in the presence of m1/2TTR-5K/5R/5H (4 μM).

Agent	T_{lag} (h)
$A\beta_{40}$	9.1 ± 2.4
TTR + $A\beta_{40}$	15.9 ± 1.9
m1TTR-5K + $A\beta_{40}$	112.3 ± 1.3
m1TTR-5R + $A\beta_{40}$	85.5 ± 1.5
m1TTR-5H + $A\beta_{40}$	71.3 ± 2.0
m2TTR-5K + $A\beta_{40}$	11.4 ± 3.0
m2TTR-5R + $A\beta_{40}$	118.8 ± 3.0
m2TTR-5H + $A\beta_{40}$	40.1 ± 1.2

Table S2. T_{lag} for amyloid formation kinetics of $A\beta_{40}$ (25 μM) incubated with different agents.

Agent	T_{lag} (h)			
	$A\beta_{40}$	+ 0.5 μM	+ 1 μM	+ 2 μM
	9.4 ± 0.3	-	-	-
TTR	-	-	10.1 ± 1.3	11.7 ± 2.5
TTR-5R	-	2.2 ± 0.3	15.0 ± 0.1	15.5 ± 0.5
TTR-7R	-	15.5 ± 0.2	29.8 ± 0.3	55.0 ± 0.3
TTR-9R	-	9.1 ± 0.4	45.6 ± 0.1	45.2 ± 0.2

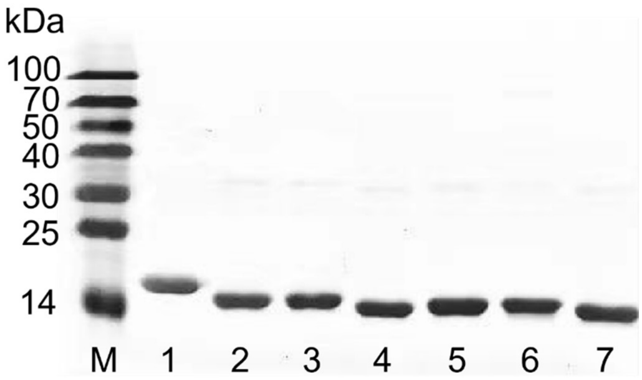

Figure S1. SDS-PAGE analysis of TTR mutated at five sites. M: Protein marker; Lane 1, TTR; Lane 2, m1TTR-5K; Lane 3, m1TTR-5R; Lane 4, m1TTR-5H; Lane 5, m2TTR-5K; Lane 6, m2TTR-5R; Lane 7, m2TTR-5H.

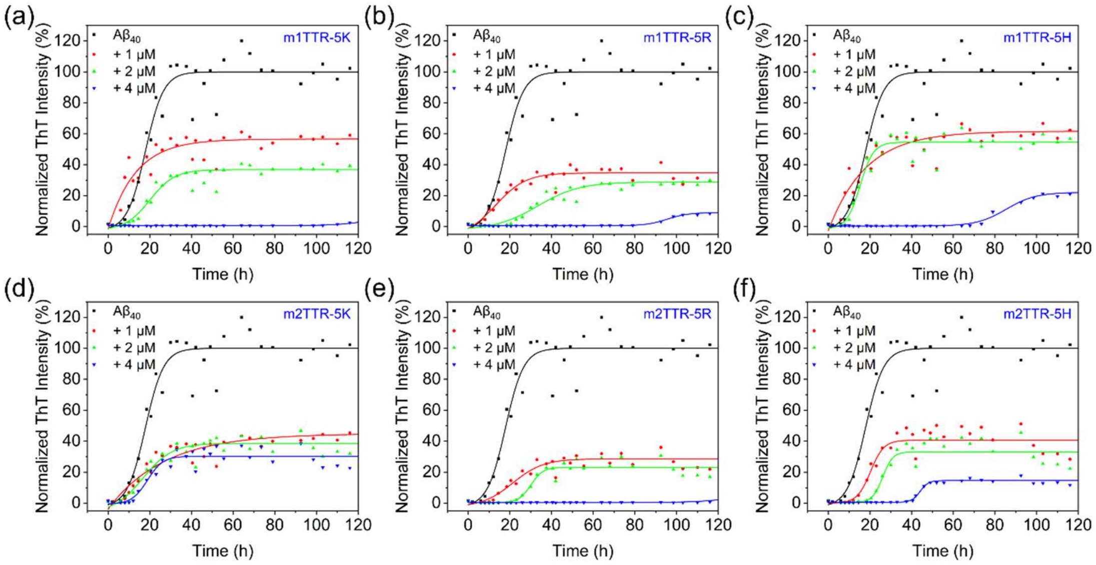

Figure S2. Effects of (a) m1TTR-5K, (b) m1TTR-5R, (c) m1TTR-5H, (d) m2TTR-5K, (e) m2TTR-5R, and (f) m2TTR-5H on Aβ_{40} aggregation measured by ThT fluorescence assay. Aβ_{40} concentration was 25 μM.

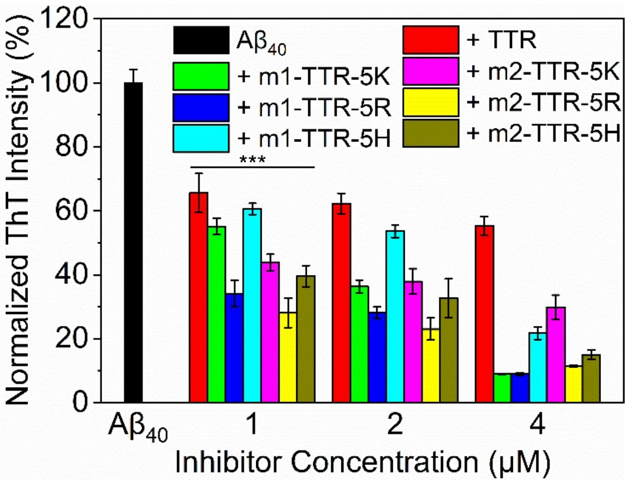

Figure S3. Final ThT fluorescence of Aβ_{40} (25 μM) incubated with different inhibitors for 120 h. ***$p < 0.001$, compared to the Aβ_{40} group.

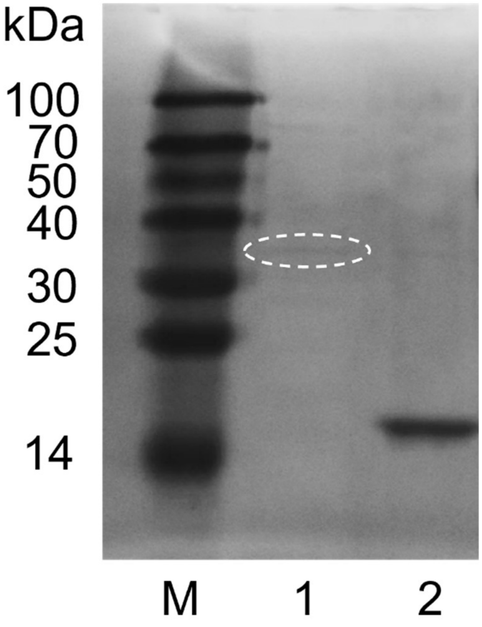

Figure S4. SDS-PAGE bands of TTR-7R and TTR-9R. M: Protein marker; Lane 1, TTR-9R; Lane 2, TTR-7R.

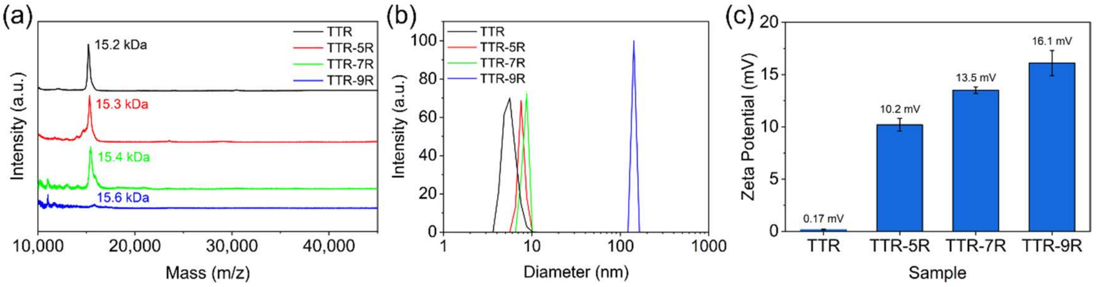

Figure S5. (a) MALDI-TOF MS, (b) size distribution, and (c) ζ-potential of TTR and TTR-5R/7R/9R.

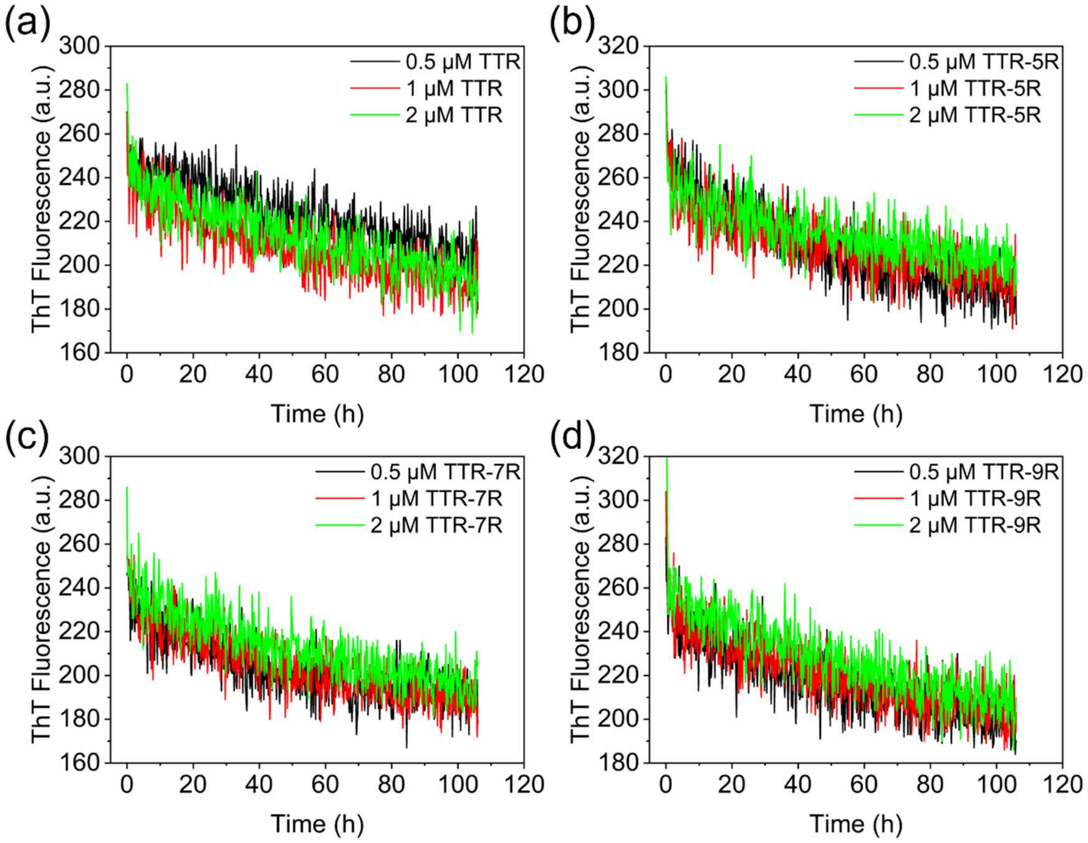

Figure S6. Time-dependent ThT fluorescence changes of (a) TTR, (b) TTR-5R, (c) TTR-7R, and (d) TTR-9R alone.

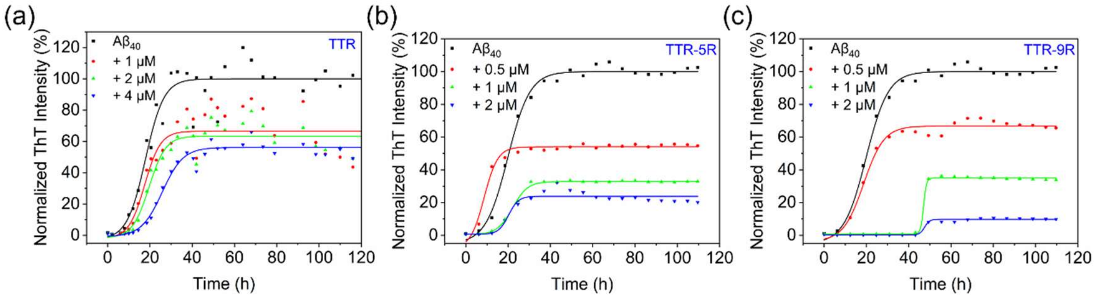

Figure S7. ThT fluorescence kinetic assay of Aβ40 incubated with (a) TTR, (b) TTR-5R, and (c) TTR-9R. Aβ40 concentration was 25 μM.

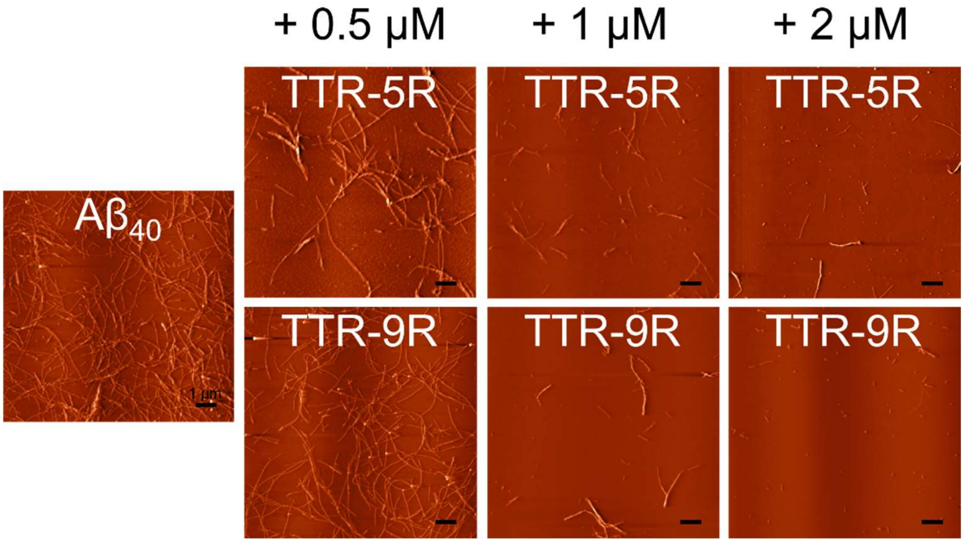

Figure S8. AFM images of Aβ40 (25 μM) aggregates alone or co-incubated with different concentrations of TTR-5R and TTR-9R. Scale bar, 1 μm.
